# Successful WATCHMAN Device Placement Despite Left Atrial Appendage Perforation in Two Cases

**DOI:** 10.7759/cureus.13251

**Published:** 2021-02-09

**Authors:** Sangeeta Prabhakar Bhat, Neil R Patel, Nishant Sethi

**Affiliations:** 1 Internal Medicine, The Wright Center for Graduate Medical Education, Scranton, USA; 2 Cardiology, The Wright Center for Graduate Medical Education, Scranton, USA; 3 Cardiology, Commonwealth Health Physician Network, Scranton, USA

**Keywords:** watchman, left atrial appendage perforation, pericardial staining, pericardial tamponade, device deployment, hemostasis

## Abstract

The WATCHMAN (Boston Scientific, Marlborough, USA) is a device used to occlude the left atrial appendage (LAA) in patients of non-valvular atrial fibrillation (NVAF) with a high CHA_2_DS_2_-VASc score who are poor candidates for oral anticoagulation. LAA perforation is a well-known complication of the WATCHMAN device placement. Here we present two cases of NVAF where oral anticoagulation was not advisable due to recurrent bleeding episodes. They underwent the WATCHMAN procedure for stroke prevention. During the placement of the WATCHMAN device into the left atrial appendage (LAA) in both cases, pericardial staining was noted that worsened over the next few minutes. It was decided to deploy the 27 mm WATCHMAN device into the LAA. In one case, satisfactory hemostasis was achieved with the device deployment eliminating the need for cardiothoracic surgery. However, the second case led to pericardial tamponade and was managed by the placement of a pericardial window. To our knowledge, this is the first case series describing the use of a WATCHMAN device upon detection of LAA perforation.

## Introduction

The most common source of thromboemboli in patients of non-valvular atrial fibrillation (NVAF) is the left atrial appendage (LAA) [1]. The WATCHMAN (Boston Scientific, Marlborough, USA) device is a left atrial appendage occlusion device used in NVAF patients with a high CHA2DS2-VASc score who are poor candidates for oral anticoagulation [2]. The device-related complications are noted to be 8.7% per the PROTECT AF (WATCHMAN Left Atrial Appendage System for Embolic Protection in Patients with Atrial Fibrillation) trial [3]. Pericardial effusion was noted in 4.5% of cases of which 3.3% required surgical intervention or pericardiocentesis [4].

## Case presentation

First case

A 76-year-old female with a past medical history of paroxysmal non-valvular atrial fibrillation on coumadin (CHA2DS2-VASc score 6, HAS-BLED score 4), stroke (that occurred seven years ago), hypertension, dyslipidemia, chronic kidney disease (stage IIIa) was evaluated as an in-patient for WATCHMAN device placement. She had developed recurrent upper gastrointestinal (GI) bleeds due to gastric ulcers and arteriovenous malformations in a diverticulum of the gastric fundus. It was decided that it was best for the patient to undergo a WATCHMAN procedure to decrease the risk of stroke. Her vitals were stable and a cardiopulmonary examination revealed an irregularly, irregular rhythm but she was otherwise mostly unremarkable.

Her laboratory tests revealed a hemoglobin level of 11.2 g/dl, and normal white blood cell and platelet counts. Her renal panel was unremarkable except for a serum creatinine of 1.6 mg/dl (normal range 0.6 to 1.1 mg/dL). Her international normalized ratio (INR) was 1.02. The initial workup included a cardiac Computed Tomography (CT) scan which revealed no filling defect in the LAA. Transesophageal echocardiography (TEE) showed a maximal orifice diameter of 23 mm. She was felt to be a candidate for the WATCHMAN occlusion device with cardiac anatomy permitting its use.

**Figure 1:**
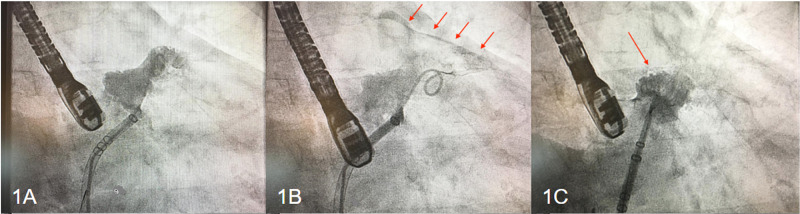
Cardiac catheterization images of the first case Cardiac catheterization images of the first case demonstrate satisfactory positioning of the pigtail catheter into the intact left atrial appendage (LAA) (1A), pericardial staining (red arrows) with the advancement of the catheter into the anterior lobe of LAA prior to WATCHMAN device placement (1B) achievement of satisfactory hemostasis after device deployment (1C).

Informed consent from the patient was obtained. Femoral vein access was gained and a 5-French angled pigtail catheter was inserted. Injection of contrast confirmed the satisfactory position of the pigtail catheter at the LAA orifice as noted in Figure 1A. Upon further advancement of the sheath, a slight staining of the pericardium was noticed, which worsened over the next few minutes (Figure 1B). To prevent further progression to frank effusion upon recognition of the leak, it was decided to go ahead with the deployment of the device (Figure 1C). Satisfactory hemostasis was obtained. TEE showed satisfactory results with trace pericardial effusion and minimal peri-device leakage. The patient tolerated the procedure well without any hemodynamic instability. The effect of heparin was reversed using protamine sulfate. After achieving hemostasis in the groins, she was extubated and transferred to the intensive care unit (ICU). She remained hemodynamically stable overnight. A limited 2D transthoracic echocardiogram performed on the next day revealed trace pericardial effusion without evidence of cardiac tamponade and she was discharged home on aspirin and coumadin with close follow-up of her hemoglobin levels. TEE 45 days after device implantation revealed a well-seated WATCHMAN occluding device with a superior leak of less than 1 mm in width. No thrombus was noted on the anterior end of the device and no pericardial effusion was visible.

Second case

An 84-year-old female with a past medical history of paroxysmal atrial fibrillation (CHA2DS2-VASc score 6, HAS-BLED score 4), essential hypertension, hyperlipidemia, chronic obstructive pulmonary disease, and a transient ischemic attack was scheduled to undergo WATCHMAN device placement due to the occurrence of recurrent iliopsoas hematomas on anticoagulation. Her vitals and systemic examination were unremarkable.

Laboratory tests revealed a hemoglobin level of 12.1 g/dl, leukocytosis with a count of 13.3 x 103/μl and a left shift, normal platelets, INR of 1.2, and an unremarkable renal function panel. Initial TEE revealed a windsock-shaped left atrial appendage with a single predominant lobe and a small meniscal secondary lobe. A CT scan of the heart did not reveal a filling defect in the LAA. The minimal appendage available for the sheath placement was more than 25 mm. A WATCHMAN device of 27 mm was determined to be of an appropriate size for placement in the patient's left atrial appendage.

**Figure 2:**
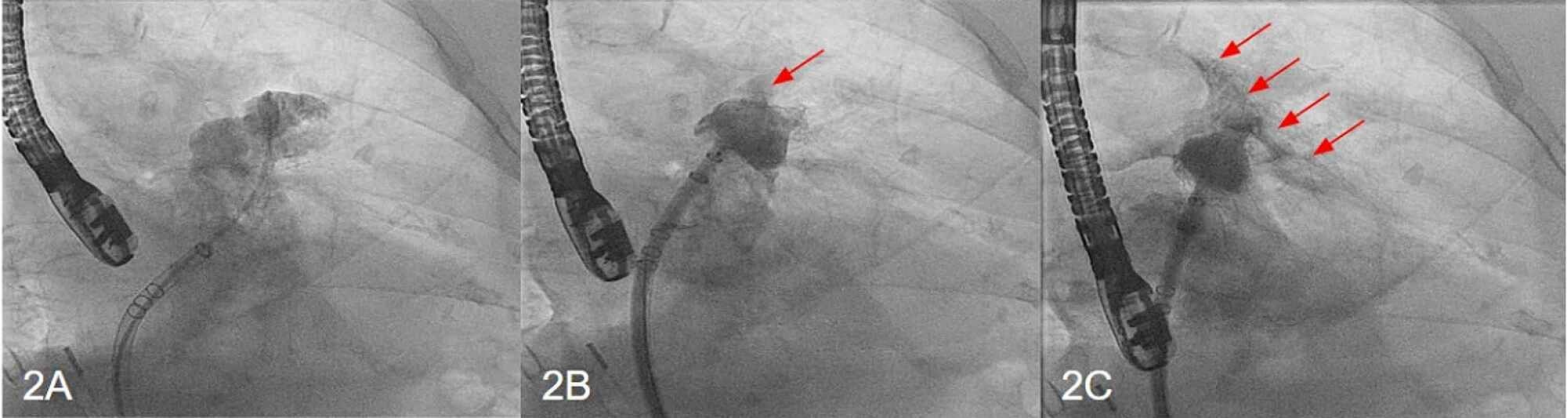
Cardiac catheterization images of the second case Cardiac catheterization images of the second case demonstrate the satisfactory positioning of the pigtail catheter into the intact left atrial appendage (2A), pericardial staining (red arrows) during WATCHMAN device deployment (2B), and worsening pericardial effusion after device deployment (2C).

After obtaining consent and femoral vein access, the sheath was advanced into the anterior lobe of the LAA. Contrast injection revealed no evidence of extravasation or effusion (Figure 2A). The WATCHMAN device was then introduced through the sheath and delivered. Early pericardial staining was noted during device deployment (Figure 2B) that gradually worsened (Figure 2C). Post-deployment TEE confirmed the development of pericardial effusion. The device was visualized and shown to be in a good position. Pericardiocentesis was performed via a percutaneous approach and was unable to successfully drain the effusion. During this procedure, the patient required intravenous fluid resuscitation and intermittent pressor support. The department of cardiothoracic surgery (CTS) was consulted for the placement of a pericardial window after the reversal of heparin. Due to persistent bleeding, the window was converted to an open drainage procedure revealing perforation of the LAA apex. The perforation was sutured and glued. The WATCHMAN device post-procedure was found to be in a stable position with an excellent seal of the LAA. Chest tubes were placed and she was transferred to the ICU. She eventually recovered after a long hospital stay of 12 days and was discharged on Coumadin 5 mg daily. A follow-up TEE, 45 days later, revealed a well-seated WATCHMAN device with no evidence of leakage. No intracavitary mass or thrombus was noted; no pericardial effusion was visible.

## Discussion

The LAA is a component of the left atrium that originates from the anterolateral primordial atrium. It is a highly crenelated structure of a variable shape, commonly categorized as a chicken wing, windsock, cactus, or cauliflower [5, 6]. In fibrillating atria, LAA is the most common source of thromboemboli due to low flow and stasis of blood. Approximately 90% of cardioembolic strokes can be attributed to thrombus in the LAA [1]. Stroke prevention can be particularly challenging in patients with a higher risk of bleeding. Over the years, several nonpharmacological approaches have been tried. Holmes et al [2] cover a brief overview of the numerous devices used for LAA occlusion including the WATCHMAN^TM^, AMPLATZER^TM^, LARIAT®, AtriClip, and TigerPaw System.

The WATCHMAN device is a novel device designed to cover the ostium of the LAA and prevent embolization. It comprises a polyester fabric and self-expanding nitinol frame structure with fixation barbs. It is available in diameters 21 to 33 mm and delivered by a catheter-based delivery system [3].

In a multicenter, randomized trial, percutaneous closure of the LAA with the WATCHMAN device was found to be non-inferior to that of warfarin therapy, the probability of non-inferiority being over 99%. However, the adverse events were higher with the intervention group as compared to the Warfarin group (RR 1·69, 1·01-3·19) owing mostly to periprocedural complications [2]. Hence, the WATCHMAN device is regarded as an alternative strategy to anticoagulation for stroke prophylaxis in NVAF patients with a high risk of bleeding.

The device-related complications were noted to be 8.7% per the PROTECT AF trial [3]. LAA is a thin-walled structure and fragile in parts [6] and can be easily perforated during WATCHMAN device placement. Pericardial effusion was noted in 4.5% of NVAF patients that underwent WATCHMAN device placement of which 3.3% required surgical intervention or pericardiocentesis [4]. The pericardial effusion was noted to prolong the hospital stay. Other procedural risks that occurred with device implantations included anesthetic-related and peripheral vascular complications, as well as early embolic events [3].

Per the multicenter EWOLUTION registry (Evaluating Real-Life Clinical Outcomes in Atrial Fibrillation Patients Receiving the WATCHMAN Left Atrial Appendage Closure Technology), 2.7% of the patients who underwent WATCHMAN device placement developed pericardial tamponade within seven days of the procedure [7]. The common mechanisms described for the development of pericardial tamponade relate to transseptal puncture or manipulation of device or delivery equipment in the left atrium or LAA during device placement [4]. According to Mobius-Winkler et al [8], pericardial effusion can be acute, subacute, or late. Certain techniques can help reduce the risk of an appendage perforation - use of TEE and pressure monitoring, use of a pigtail catheter or looped wire to guide the access sheath into the LAA, slow and careful device manipulation, and tug-testing after device implantation. These techniques are often used collectively but the efficacy of each preventive technique remains undetermined. Further analysis of data from the EWOLUTION study speculated that sinus rhythm increases the risk of pericardial effusion and tamponade during WATCHMAN placement but the multivariate analysis did not identify it as an independent predictor of complications [9].

Although there is sufficient literature on complications during device placement, there are no guidelines or protocols to suggest the management of the same. In the first case, LAA perforation occurred prior to the device placement, and in the second case, it occurred during the device placement. In the above cases, the decision of immediate deployment of the device was a difficult one. We believe that minimally invasive methods are always preferred prior to major surgical interventions. Further studies are required to determine the outcome of device placement in cases where periprocedural pericardial staining is noted.

## Conclusions

In the first case, pericardial effusion occurred prior to the device deployment and could be fully managed by device placement resulting in a short hospital stay. However, the second case was complicated by pericardial effusion during device placement resulting in tamponade. The patient required CTS intervention and a prolonged hospital stay. Both cases eventually had successful WATCHMAN device implants on follow-up TEE. This case series reflects upon the management of pericardial staining by the deployment of the WATCHMAN device in an attempt to prevent progression to large pericardial effusion/tamponade, hemodynamic instability, or the need for major cardiothoracic surgery. If and when device deployment is unable to control the pericardial effusion, cardiothoracic surgery should be considered for further management.
